# Artificial nighttime light changes aphid-parasitoid population dynamics

**DOI:** 10.1038/srep15232

**Published:** 2015-10-16

**Authors:** Dirk Sanders, Rachel Kehoe, Katie Tiley, Jonathan Bennie, Dave Cruse, Thomas W. Davies, F. J. Frank van Veen, Kevin J. Gaston

**Affiliations:** 1 Centre for Ecology & Conservation, School of Biosciences, University of Exeter, Cornwall Campus, Penryn, Cornwall, TR10 9EZ, United Kingdom; 2 Environment & Sustainability Institute, University of Exeter, Cornwall Campus Penryn, Cornwall, TR10 9EZ, United Kingdom

## Abstract

Artificial light at night (ALAN) is recognized as a widespread and increasingly important anthropogenic environmental pressure on wild species and their interactions. Understanding of how these impacts translate into changes in population dynamics of communities with multiple trophic levels is, however, severely lacking. In an outdoor mesocosm experiment we tested the effect of ALAN on the population dynamics of a plant-aphid-parasitoid community with one plant species, three aphid species and their specialist parasitoids. The light treatment reduced the abundance of two aphid species by 20% over five generations, most likely as a consequence of bottom-up effects, with reductions in bean plant biomass being observed. For the aphid *Megoura viciae* this effect was reversed under autumn conditions with the light treatment promoting continuous reproduction through asexuals. All three parasitoid species were negatively affected by the light treatment, through reduced host numbers and we discuss induced possible behavioural changes. These results suggest that, in addition to direct impacts on species behaviour, the impacts of ALAN can cascade through food webs with potentially far reaching effects on the wider ecosystem.

The introduction of artificial lighting into the nighttime environment arguably constitutes one of the most profound environmental pressures to have been exerted by humans. Such lighting derives from a diversity of sources (including street lighting) and has already become widespread^1^. It is continuing to spread rapidly^2^, and interacts and synergises with many other environmental pressures^3^. Artificial light at night (ALAN) constitutes an evolutionarily novel anthropogenic environmental pressure for which there have been no natural analogues^3^; natural light cycles have remained largely stable for millennia, allowing “light guided” biological responses to become evolutionarily and phylogenetically deep rooted.

Large numbers of studies have documented the effects of ALAN on organismal physiology and behaviour^4^. These responses have repeatedly been predicted to impact population dynamics of species^5^. However, empirical studies, and especially experimental ones, largely remain wanting^6^,^7^. Studies of the impacts of ALAN on population dynamics would particularly benefit from (i) using established model systems; (ii) running experiments for multiple generations; (iii) examining multiple trophic levels; and (iv) determining effects on the numbers of organisms that are not simply a consequence of organismal movements into or away from the area that is lit^5^. Here we report the results of an experimental study of the impacts of ALAN on a multi-species plant-aphid-parasitoid system.

Plant-aphid-parasitoid systems are used widely as models in ecological research because of their abundance and importance in most temperate terrestrial ecosystems and the tractability of host-parasitoid interactions. There are several reasons to predict that such systems will be susceptible to ALAN. First, studies have shown that the life cycles of aphids are sensitive to changes in light regime, with the length of the photoperiod an important influence that either increases or decreases fecundity depending on species and factors such as light intensity and temperature^8^. Under spring/summer conditions aphids reproduce asexually but can switch to sexual reproduction in autumn (a strategy preferable under harsh winter conditions), again under the influence of the reduction in length of the daily photoperiod^9^. Second, it is also known that many flying insects change their behaviour when exposed to ALAN^10^, and there is evidence that changing the photoperiod also affects fecundity in parasitoids (e.g.^11^). Third, aphids and their parasitoids are not only common in many temperate habitats, but also on vegetation in urban, agricultural and natural ecosystems exposed to varying amounts of ALAN.

In an outdoor mesocosm experiment with a nighttime lighting treatment and unlit control we tested for the effect of ALAN on the population dynamics of aphids and associated parasitoids, and on aphid host plant biomass, using a community with one plant species and three aphid species and their specialist parasitoids^12^,^13^. We predicted that (1) ALAN would decrease aphid population sizes, as this appeared to be the most likely outcome for temperatures below 20 °C and an extended photoperiod^8^, and (2) the production of aphid sexuals and the number of eggs laid under autumn conditions would be reduced by increasing the perceived daylength. Further, (3) we predicted a negative impact of artificial light on parasitoids by reducing their population size as a consequence of reduced host numbers.

## Results

### Aphids

**Figure 1:**
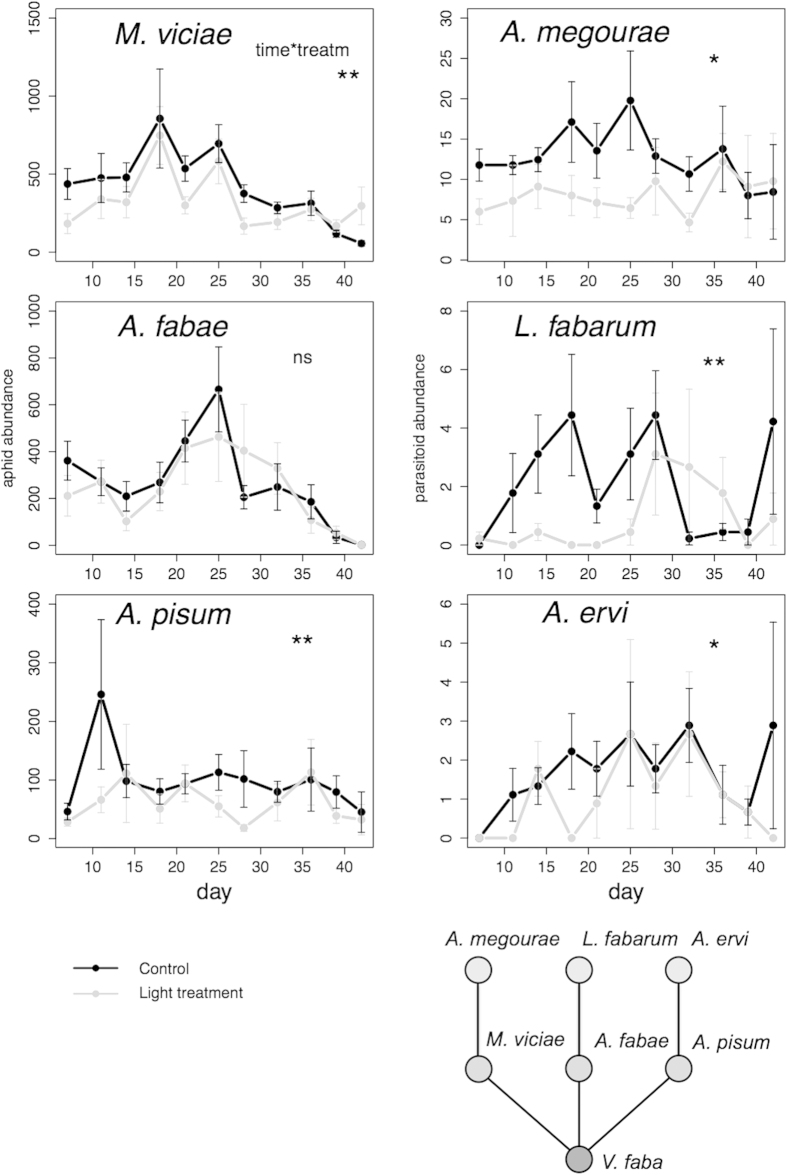
Population dynamics (means of 9 replicates + standard error) of the aphid species *Megoura viciae*, *Aphis fabae* and *Acyrthosiphon pisum* each attacked by a specialist parasitoid namely *Aphidius megourae*, *Lysiphlebus fabarum* and *Aphidius ervi*, in control (black) and light treatment (grey) cages. *p < 0.05 and **p < 0.01, indicate a significant effect of the light treatment and in case of *M. viciae* an interaction between treatment and time on abundance tested with linear mixed effects models.

**Figure 2:**
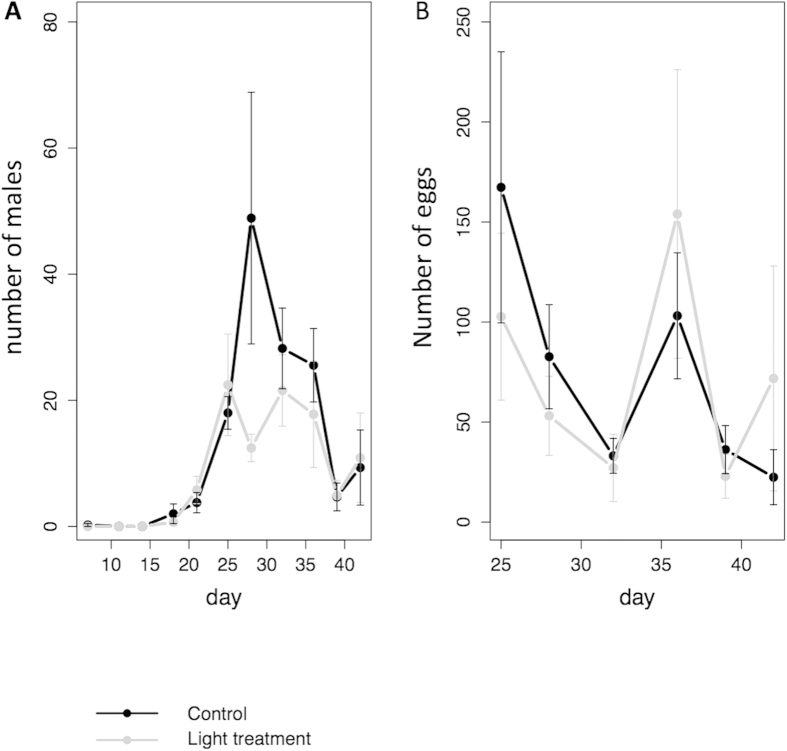
(**A**) Numbers of males and (**B**) eggs of the aphid *Megoura viciae*, (means of 9 replicates + standard error) in control (black) and light treatment (grey) cages.

Over the course of the experiment (approx. 5 aphid generations) total aphid abundance (summing across species) was on average reduced by 20% in the light treatment, an effect that changed over time (treatment x time interaction: L. Ratio = 7.25, p =0 .0071). The dominant aphid *M. viciae* and its parasitoid *A. megourae* showed a remarkably quick response to the light treatment with differences in abundance already visible a few days after the lights were switched on (Fig. 1). The negative effect on *M. viciae* was reversed towards the end of the experiment (time x treatment interaction L. ratio = 8.58, p = 0.0034). Light treatment had no effect on numbers of males and egg production in *M. viciae* (Fig. 2, L. ratio =  1.36, p = 0.2435 ; L. ratio = 1.67, p =  0.1968, respectively). *A. pisum* was also negatively influenced by light treatment (L. ratio = 9.55 , p = 0.002) while there was no effect on *A. fabae* (L. ratio = 2.06, p = 0.1512).

### Parasitoids

**Figure 3:**
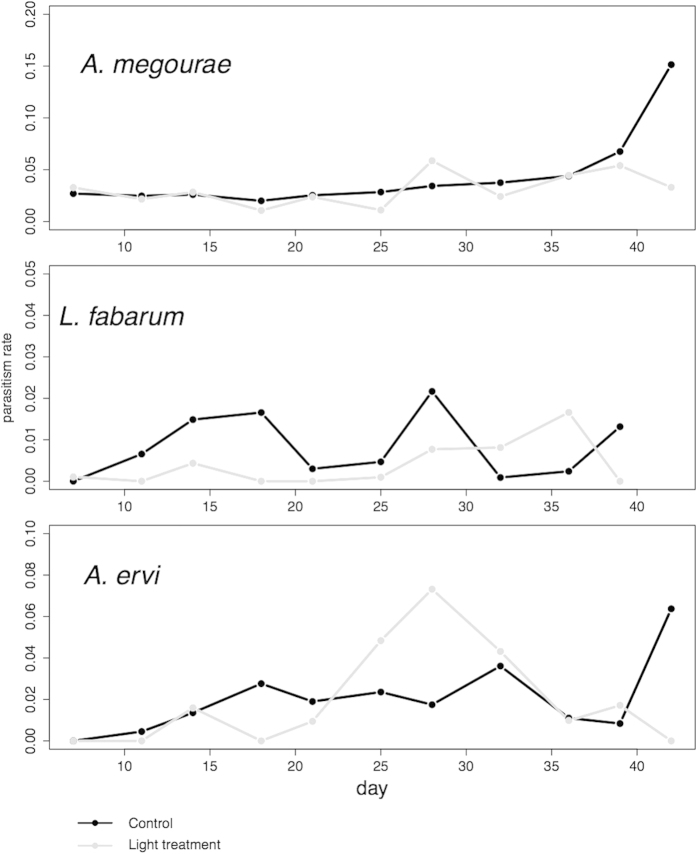
Parasitism rate for the parasitoids *Aphidius megourae*, *Lysiphlebus fabarum* and *Aphidius ervi* in control (black) and light treatment (grey) cages. For *L. fabarum* no data were available for the last count as the host *A. fabae* had gone extinct in most replicates.

During the experiment (4 parasitoid generations) overall parasitoid abundance declined by 40% in the light treatment (L. Ratio = 4.27, p = 0.0388). *A. megourae* followed the pattern of its host, with a strong negative impact of light treatment in the beginning that was less obvious toward the end (L. ratio = 4.56, p = 0.0327, Fig. 1). *L. fabarum* was very strongly affected by light treatment in the first three weeks (L. ratio = 7.12, p = 0.0076) despite there being no population response of its host to the treatment. *A. ervi* was similarly to the other parasitoid species, but less strongly, affected by artificial light (L. ratio = 5.17, p = 0.023). Parasitism rate for *L. fabarum* dropped from 0.8% in the control to 0.4% in the light treatment (Fig. 3, z = –2.81, p = 0.00501), while there was no significant change for *A. megourae* and *A. ervi* (Fig. 3, z = −0.80, p = 0.42; z = −0.38, p =  0.70, respectively).

### Plant biomass

Plant biomass was not affected by aphid abundance. After the removal of two outliers (unusual high plant biomass) in the dataset, light treatment reduced mean aboveground plant biomass from 1.46 g (±0.2 SE) in the control treatment to 0.99 g (±0.1) per plant pot (L. Ratio = 6.78, p =  0.0092). In the follow on experiment testing the impact of artificial light on plants without aphids, plant biomass was similarly reduced from 13.5 g ± 0.4 in the control cages to 11.7 g ± 0.5 in light treatment cages (L. Ratio = 7.33, p =  0.0068).

## Discussion

With the exception of work on a microbial and on another insect community^6^,^7^, this is, in terms of generations, the longest running experiment on the population effects of ALAN to have been reported to date. Strong negative effects of ALAN on abundances were found both for aphids and their parasitoids (Fig. 1). Indeed, for the aphid *M. viciae* and its parasitoid these responses were observed surprisingly quickly (within one week) and sustained until close to the end of the experiment when the impact of the light treatment on population size switched to positive by promoting the continuous production of asexuals.

The responses of animal populations to ALAN can potentially result from a direct impact (e.g. growth rate or production of sexuals) or indirectly through bottom-up (resource controlled), top-down (predator or parasite), or non-trophic (e.g. competition) processes^7^. For the aphid species, declines in numbers as a consequence of ALAN in the experiments reported here most likely arose from bottom-up effects, with reductions in bean plant biomass and plant quality due to light treatment reducing the resource available to these herbivores. Low intensity light at night, at levels similar to those used in this study, can alter resource allocation and growth form in plants in complex ways, including suppressing growth^14^. In a previous study^7^, bottom-up effects of ALAN on an aphid population were likely driven by supressing flowering in their leguminous foodplant and decreasing the abundance of resources. The simultaneous negative effect of the ALAN treatment on parasitoid numbers makes it unlikely that any top-down effect could be operating in this study by an increased attack rate leading to lower aphid numbers. For *M. viciae*, populations subjected to ALAN became more abundant relative to the controls towards the end of the experiment (Fig. 1). This is a consequence of the continuous production of asexuals under ALAN, showing that this can alter the normal seasonal strategy of switching to sexual reproduction in the autumn. This is the first time that ALAN, similar to the effect of extending the photoperiod, has been shown to affect not simply the timing of reproduction by an animal species but this form of reproduction. However, this apparently positive effect on reproduction means that these aphids will not survive the winter condition as they feed on annual plants and only sexually produced eggs are frost hardy.

That there was no apparent population response in the aphid *A. fabae* to the light treatment does not necessarily mean that there was no negative effect as this might have been compensated by a release from competition by the dominant aphid *M. viciae*.

The negative impact of ALAN on parasitoid numbers in the experiment is unlikely to be a bottom-up effect only. The numbers of potential aphid hosts remained high throughout the experiment relative to the numbers of parasitoids, and in the case of *A. fabae* and *L. fabarum* there was a negative impact of ALAN on parasitoid numbers that was not observed in its host (Fig. 1). This suggests that ALAN interferes with parasitoid searching behaviour, fecundity, or host resistance leading to fewer successful attacks on aphids.

While the impact of nighttime lighting on aphid carrying capacity and reduced parasitism rate and parasitoid numbers are likely to be observed under spring and summer conditions, the effect on the reproduction of sexuals and eggs in *M. viciae* will only occur in autumn, when it naturally switches to sexual reproduction.

Our experiment demonstrated the potential for street, and other forms, of artificial nighttime lighting to affect multiple trophic levels in communities at the same time, with potentially upward and downward cascading effects. This begs the question of the extent to which effects of ALAN are under-recorded through an almost exclusive focus to date on direct effects^7^.

## Methods

### Experiment

The plant-aphid-parasitoid communities consisted of bean plants (*Vicia faba*, L., var. the Sutton) as the food resource for the three aphid species *Aphis fabae* (Scopoli), *Acyrthosiphon pisum* (Harris) and *Megoura viciae* (Buckton), each attacked by a specialist parasitoid, namely *Lysiphlebus fabarum* (Marshall), *Aphidius ervi* (Haliday) and *Aphidius megourae* (Stary), respectively. In the absence of systematic environmental pressures, this community is very stable due to positive indirect interactions between the parasitoid species that promote coexistence^13^.

These communities were established in 30 × 30 × 60 cm wooden framed mesocosms with thrip netting (mesh size  = 0.15 × 0.49 mm) in an outdoor field site at the University of Exeter’s Penryn Campus. In each mesocosm, insect communities were established by placing 5 adult aphids of each species on 2 pots each with four 2 week old bean plants, on 18. 08. 2014. Aphids were introduced from a stock culture from a greenhouse, set at 20 ^o^C, with no artificial lighting. After both one and two weeks, 2 mated adult female parasitoids of each species were added to each cage, alongside 2 pots with fresh plants. Each week, 2 pots with “2 week old” plants were added to each cage, with the oldest pots being removed, whilst keeping all insects in the cage. This procedure allowed the observation of generationally long-term population dynamics^13^.

The light treatment and control were replicated 9 times and arranged in a block design, with a 1 m distance between cages (sufficient to avoid treatment replicates influencing controls): lit and non-lit treatments were paired, and located next to each other. The light treatment cages had a ‘cool white’ LED strip fixed to the ceiling of the cage, surrounded by wooden slats to prevent the over-spilling of light, which was switched on after the communities were established (08. 09. 2014). A light intensity within treatment cages of approximately 30 lux was selected to represent typical levels measured in low-growing roadside vegetation beneath streetlights (typically between 10 and 40 lux). The spectrum of the light sources was identical to that used in other mesocosm experiments to study the impacts of artificial nighttime lighting^7^, and is similar to that in commercial LED street lighting systems, with a peak in the blue portion of the spectrum (around 445 nm) and a broad secondary peak between around 500 and 650 nm. A light sensor caused the lights to come on at dusk (below 70 lux), and off at dawn (below 110 lux) in the light treatment cages.

From the third week after the introduction of the parasitoids, twice weekly the number of every species (aphids and aphid mummies), including the number of sexual aphids and aphid eggs, were counted. In the second to last week of the experiment, the oldest plants were removed, washed, air-dried at 65 °C for 2 days, and weighed. To test for the impact of artificial light on bean plants without any aphids we conducted a follow on experiment with two pots with four two week old bean plants per cage in the same cages as the main experiment. This additional experiment ran from 14^th^ to 28^th^ May 2015. Plant material was handled in the same way as in the main experiment.

### Statistical analysis

We tested the impact of the light treatment on the six species and aphid egg numbers (for *M. viciae* only) using linear mixed effects models with treatment x time (counting events) as fixed factors, while including replicate (cage identity) nested in block as a random factor. To account for systematic trends over time we also included time squared as a covariate. Because the residuals showed a significant partial temporal autocorrelation, we included a first-order autoregression in the models. Analyses were performed with the nlme package^15^ in R 3.1.0^16^. Significance levels for treatment effects were determined by removing factors from the model and likelihood ratio tests. Response variables (egg number, aphid and parasitoid abundance) were log or square root transformed to meet model assumptions. For testing for the impact on aphid eggs we had data from week 3 onwards, when we first observed egg production. The impact on parasitism rate was analysed using generalised linear mixed models assuming a binomial error distribution. The dependent variable was the bivariate variable containing ‘mummies of host i’ and ‘numbers of host i’, where ‘i’ is one of the aphid species. Fixed and random factors were included in the same way as above. We used an analysis of covariance to test for the impact of treatment and aphid numbers (cumulative aphid numbers per mesocosm of last two counts) on plant biomass and only of treatment for the follow-on experiment on plant biomass without aphids.

## Additional Information

**How to cite this article**: Sanders, D. *et al*. Artificial nighttime light changes aphid-parasitoid population dynamics. *Sci. Rep*. **5**, 15232; doi: 10.1038/srep15232 (2015).
